# Exploring Weak Ligand–Protein Interactions by Long-Lived NMR States: Improved Contrast in Fragment-Based Drug Screening**

**DOI:** 10.1002/anie.201404921

**Published:** 2014-09-04

**Authors:** Roberto Buratto, Daniele Mammoli, Elisabetta Chiarparin, Glyn Williams, Geoffrey Bodenhausen

**Affiliations:** Institut des Sciences et Ingénierie Chimiques, Ecole Polytechnique Fédérale de LausanneBatochime (BCH), 1015 Lausanne (Switzerland); Département de Chimie, Ecole Normale Supérieure24 rue Lhomond, 75231 Paris Cedex 05 (France); Astex Pharmaceuticals, 436 Cambridge Science ParkMilton Road, Cambridge CB4 0QA (UK)

**Keywords:** dissociation constants, drug discovery, fragment screening, ligand binding, NMR spectroscopy

## Abstract

Ligands that have an affinity for protein targets can be screened very effectively by exploiting favorable properties of long-lived states (LLS) in NMR spectroscopy. In this work, we describe the use of LLS for competitive binding experiments to measure accurate dissociation constants of fragments that bind weakly to the ATP binding site of the N-terminal ATPase domain of heat shock protein 90 (Hsp90), a therapeutic target for cancer treatment. The LLS approach allows one to characterize ligands with an exceptionally wide range of affinities, since it can be used for ligand concentrations [L] that are several orders of magnitude smaller than the dissociation constants *K*_D_. This property makes the LLS method particularly attractive for the initial steps of fragment-based drug screening, where small molecular fragments that bind weakly to a target protein must be identified, which is a difficult task for many other biophysical methods.

Over the last decade, fragment screening has emerged as a powerful way to identify new lead compounds,[1] and fragment-based drug discovery (FBDD) has gained wide acceptance in pharmaceutical industry, as evidenced by a significant number of fragments that have been developed into lead series and clinical candidates.[1a, 2] For this purpose, relatively small libraries of carefully chosen compounds with low molecular weights (120–250 Da) are screened to identify fragments that can weakly bind to a protein target. Useful fragments typically have dissociation constants *K*_D_ ranging from 0.1 to 10 mm or greater. Structural biology is usually employed to establish their binding mode and guide their optimization. Techniques that can detect ligand–protein complexes, such as X-ray crystallography, surface plasmon resonance (SPR), isothermal titration calorimetry (ITC), and high-concentration assays can be used for fragment screening. The output of these target-based methods depends on the fraction of bound protein with respect to the total protein concentration.[3] If the binding affinities are weak, the equilibrium can only be shifted by increasing the concentration of the fragments, which must therefore be highly soluble, a requirement that is difficult to meet.

In ligand-based methods the output is given by the fraction of bound ligands with respect to total ligand concentration.[3] So despite its low intrinsic sensitivity, the detection of ligands by NMR spectroscopy can be used over an extremely wide dynamic range of dissociation constants *K*_D_ while requiring only relatively low protein and ligand concentrations.[3] In contrast to the above-mentioned biophysical techniques, one can perform screening with ligand concentrations [L] that are orders of magnitude lower than the corresponding dissociation constants *K*_D_. Among the best-known NMR methods, one should mention the transfer of magnetization from the solvent to protein-bound ligands (“Water-LOGSY”),[4] magnetization transfer from the protein to the ligand by saturation transfer difference (STD),[5] the accelerated transverse relaxation of ^1^H or ^19^F nuclei attached to bound ligands measured by Carr–Purcell–Meiboom–Gill (CPMG) spin echo sequences, and selective measurements of relaxation rates of ^1^H nuclei of bound ligands.[6] These methods exploit a difference in relaxation rates between bound and free ligands [Eq. (1)].

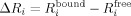
(1)

Here *i*=1 stands for longitudinal relaxation, *i*=2 for transverse relaxation, *i*=1*ρ* for relaxation in the rotating frame, *i*=LLS for long-lived states, *i*=LLC for long-lived coherences, etc. The difference Δ*R_i_* determines the observed relaxation rate 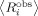, which in the fast-exchange regime[7] (provided the exchange rates are faster that the difference in chemical shifts) is a weighted average of 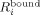 and 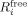 determined by the molar fractions [Eq. (2)].

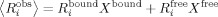
(2)

The larger Δ*R_i_*, the smaller the molar fraction *X*^bound^=[PL]/[L] that can be detected. The experimental conditions must be chosen to yield a sufficiently large contrast *C_i_* [Eq. (3)].

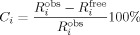
(3)

It has been proposed recently that LLS can be used for ligand–protein screening.[8] The difference Δ*R*_LLS_ can be much larger than Δ*R*_1_, Δ*R*_2_, etc., so that it is possible to achieve a high contrast *C*_LLS_ even for high ligand/protein ratios, making LLS-based screening particularly attractive for a fragment-based approach that seeks to identify weakly binding ligands.

LLS are nuclear spin states that are immune to dipole–dipole interactions between the two spins involved.[9] As their name implies, LLS have the property that their magnetization decays with a low rate constant *R*_LLS_ that is often much smaller than the longitudinal relaxation rate *R*_1_. Ratios *R*_1_/*R*_LLS_ up to 37 have been measured for pairs of protons,[10] making LLS valuable probes to study slow diffusion[11] and slow exchange phenomena[12] and to preserve hyperpolarization induced by DNP.[13] It has been shown that even a small change of the chemical shifts of the nuclei that carry the LLS upon binding can boost 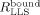, and hence increase the contrast *C*_LLS_, since the radio-frequency (rf) field that must be applied to sustain the LLS becomes inefficient if it is not applied exactly halfway between the two chemical shifts. The combination of a large contrast *C*_LLS_ and a slow relaxation rate 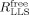

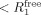
 makes the dynamic range of LLS screening particularly attractive, since one can detect the binding of fragments with dissociations constants *K*_D_ that cover a wide range. In this work we demonstrate that it is possible to measure dissociation constants up to 12 mm, where all other known biophysical techniques fail, including conventional NMR methods based on the observation of ligands.

To illustrate the advantages of LLS screening, we determined the contrast *C*_LLS_ for the LLS signals of the aromatic protons *I* and *S* of vanillic acid diethylamide (ligand II), represented by bold red letters **H** in Figure 1, during a titration against the N-terminal ATPase domain of heat shock protein 90 (Hsp90). Excitation of long-lived states (LLS) was achieved as described by Sarkar et al.[10] Three nonselective “hard” pulses 90°−*τ*−180°−*τ*−45° were used to generate zero-quantum coherences and 2*I_z_S_z_* terms. A monochromatic continuous-wave (CW) radio-frequency field (rf) was applied during a delay Δ exactly halfway between the two chemical shifts[8] to render the two spins *I* and *S* magnetically equivalent. When this rf field is effective, the dipolar interaction between spins *I* and *S* does not contribute to the LLS relaxation, hence 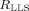

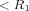
. Finally, two more pulses 45°−*τ*−180°−*τ* convert the LLS into observable *I_y_* and *S_y_* terms.

**Figure 1:**
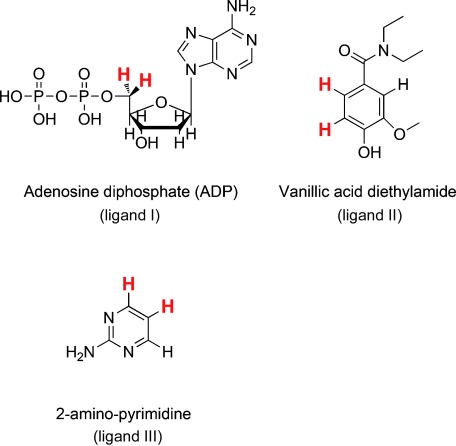
Weakly binding ligands for the ATP binding site of heat shock protein 90 (Hsp90). Pairs of protons that are suitable for the excitation of LLS are indicated by bold red letters.

Table 1 shows mole fractions *X*^bound^ of bound ligands for different ligand/protein ratios and the corresponding contrast *C*_LLS_. Even for a large ligand-to-protein ratio [L]_tot_/[P]_tot_=272, one observes a dramatic 45 % contrast.

**Table 1:** Molar fractions of bound ligands for different ligand/protein ratios and experimentally observed contrast *C*_LLS_ for a titration of vanillic acid diethylamide (ligand II, *K*_D_=790 μm) in the presence of the protein Hsp90.

[L]_tot_/[P]_tot_	*X*^bound^ [mol %]	Contrast *C*_LLS_ [%]
56	0.74	72
125	0.49	63
202	0.36	54
272	0.28	45
366	0.22	41
548	0.16	29
707	0.13	23

A contrast *C*_LLS_=23 %, corresponding to a ratio *R*^obs^/*R*^free^=1.3 could be achieved with a ratio [L]_tot_/[P]_tot_=707, in other words, under conditions where less than 0.2 % of the ligand was bound to the protein. Compared to other ^1^H-detected NMR methods, which suffer from lower contrast, this method allows ligand binding to be detected for low protein concentrations and/or low binding affinities. One can thus more easily adjust the concentrations of proteins and ligands to study very weak affinities in screening assays. For example, to detect ligands with *K*_D_≤1 mm and [L]=500 μm, one would require a protein concentration [P]=3 μm; alternatively, if [P]=20 μm one can detect binding if *K*_D_>10 mm. Such weak affinities are typically encountered for fragments that bind protein–protein interfaces. This offers considerable advantages over fragment screening by traditional ligand-based NMR methods.

Long-lived states (LLS) can be best excited within isolated two-spin systems, although larger spin systems can also support LLS.[14] Many small fragments contain suitable pairs of ^1^H or ^19^F nuclei. LLS screening is most effectively run in competition mode, as proposed by Dalvit et al.[15] for traditional *R*_1_ and *R*_2_ experiments: a strongly binding ligand partly displaces a weakly binding “spy” ligand from the binding site, so that one observes a decrease of the relaxation rate *R*_LLS_ of the displaced spy ligand. Thus, it is possible to determine the affinity of strongly binding ligands by monitoring the rate *R*_LLS_ of a spy ligand. Furthermore, by keeping the concentration of the spy ligand low, one can study competing ligands with limited solubility. This is a major advantage not only for screening mixtures, but also for determining the affinities of weakly binding fragments.

Prior to starting a fragment screening campaign, a small number of fragments are typically screened using different NMR methods. The NMR assay can then be customized to a specific protein target. During this phase a number of hits may be identified so that one can select a spy ligand that is suitable for competitive screening. To show the applicability of the method, we measured relaxation rates *R*_LLS_ in a group of three ligands that were known to bind Hsp90 in its ATP binding site, located in the N-terminal ATPase domain of the protein.[16] Adenosine diphosphate (ADP, ligand I) is the product of the ATPase reaction. Vanillic acid diethylamide (ligand II) and 2-aminopyrimidine (ligand III) have been identified as weakly binding ligands by fragment screening.[16]

LLS experiments were performed by focusing attention on pairs of scalar-coupled protons in these three compounds (in bold red letters in Figure 1). The two diastereotopic protons H5 and H5′ of the ribose group were selected to excite LLS in ADP, while pairs of aromatic protons were selected for compound II (for ligand III, we could not excite any LLS with a useful ratio *R*_1_/*R*_LLS_>1). To measure the rates 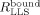, direct titration curves were measured for 0.3 mm<[L]_tot_<6 mm in the presence of [P]_tot_=10 μm Hsp90. The curves were fitted to the following function[17] given in Equation (4) to extract *K*_D_ and 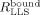.

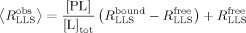
(4)

Here the ratio [PL]/[L]_tot_ is a function of the dissociation constant *K*_D_.[17] Table 2 also gives dissociation constants *K*_D_ determined by isothermal titration calorimetry (ITC)[16] for comparison.

**Table 2:** Dissociation constants *K*_D_ determined by LLS and ITC, and rates *R*_LLS_ for bound and free ligands I and II in the presence of Hsp90.

Ligand	*K*_D_ [μm] LLS	*K*_D_ [μm] ITC	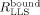 [s^−1^]	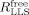 [ms^−1^]
I	15±10	10	77±6	731±7
II	708±97	790	94±3	228±11
III^[a]^	–	>1000	–	–

[a] For ligand III no useful LLS signal could be observed.

The equilibrium constants determined by LLS and ITC are in fair agreement. In order to detect fragments with 100 μm<*K*_D_<10 mm in competitive binding experiments, the weakest ligand (ligand II) that could be identified by ITC was chosen as a spy molecule. Figure 2 shows the LLS signals of ligand II in the absence (top) and in the presence (middle) of Hsp90. When Astex’s clinical Hsp90 inhibitor AT13387 is added (bottom), the signal is almost completely restored, demonstrating that both ligand II and the high-affinity inhibitor bind Hsp90 to the same ATP binding site.

**Figure 2:**
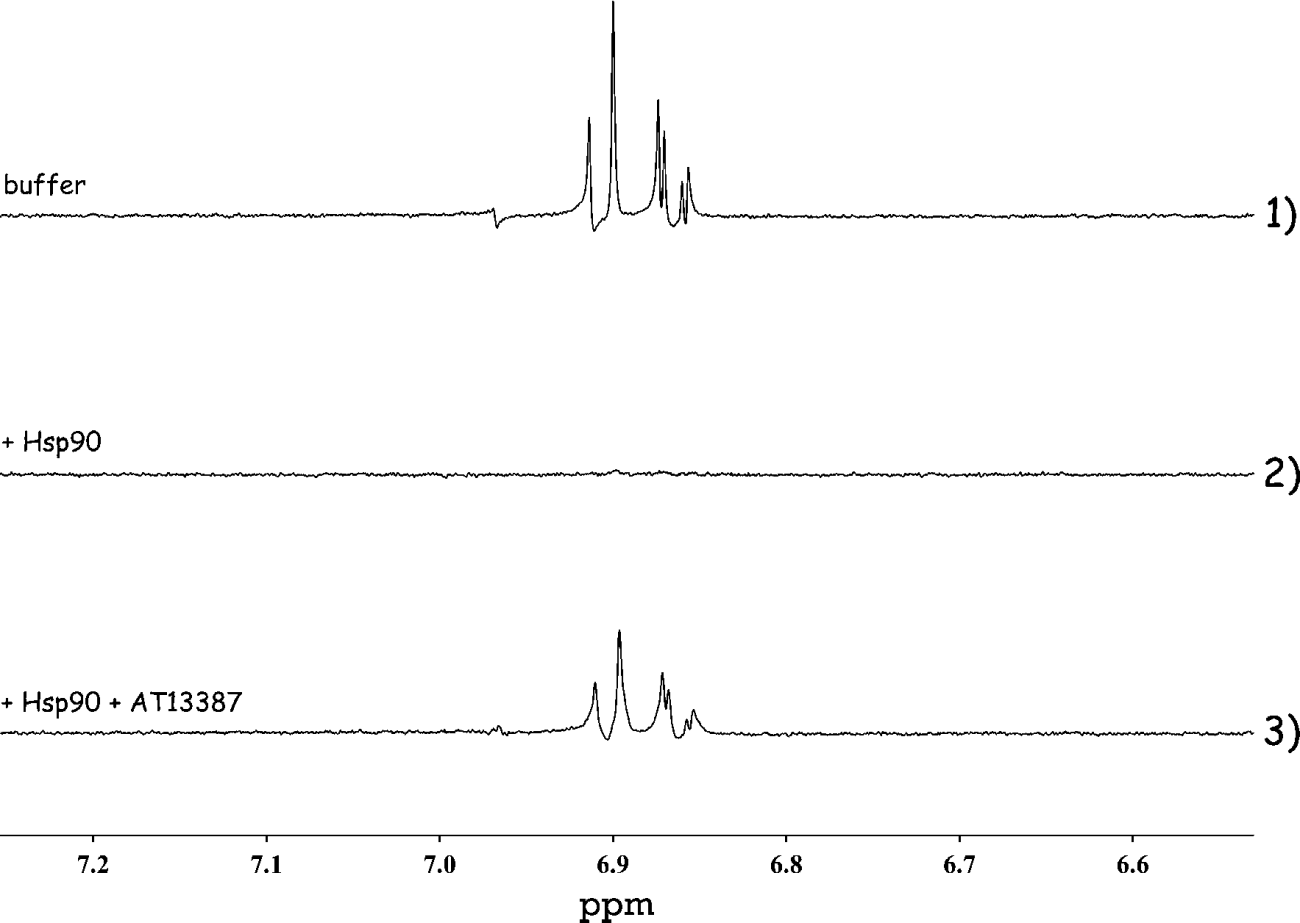
LLS NMR spectra of ligand II in three different solutions, sustaining the LLS for a delay Δ=2.5 s: 1) 500 μm ligand II in the absence of Hsp90; 2) 500 μm ligand II in the presence of 10 μm Hsp90; 3) 500 μm ligand II in the presence of 10 μm Hsp90 and 10 μm AT13387. In the latter case, ligand II is partly expelled from the ATP binding site of Hsp90 so that its LLS signal is partly restored.

If a library of, say, 1000 compounds is to be screened against a protein target, it is most efficient to screen “cocktails” containing typically three to ten ligands, to reduce experimental time and protein consumption. We tested the performance of LLS screening in competition mode with a mixture containing known binders and known nonbinders. Ligands V, VI, and VII (see Figure 3) had previously been identified as weak ADP-competitive binders during a screening campaign at Astex.[16]

**Figure 3:**
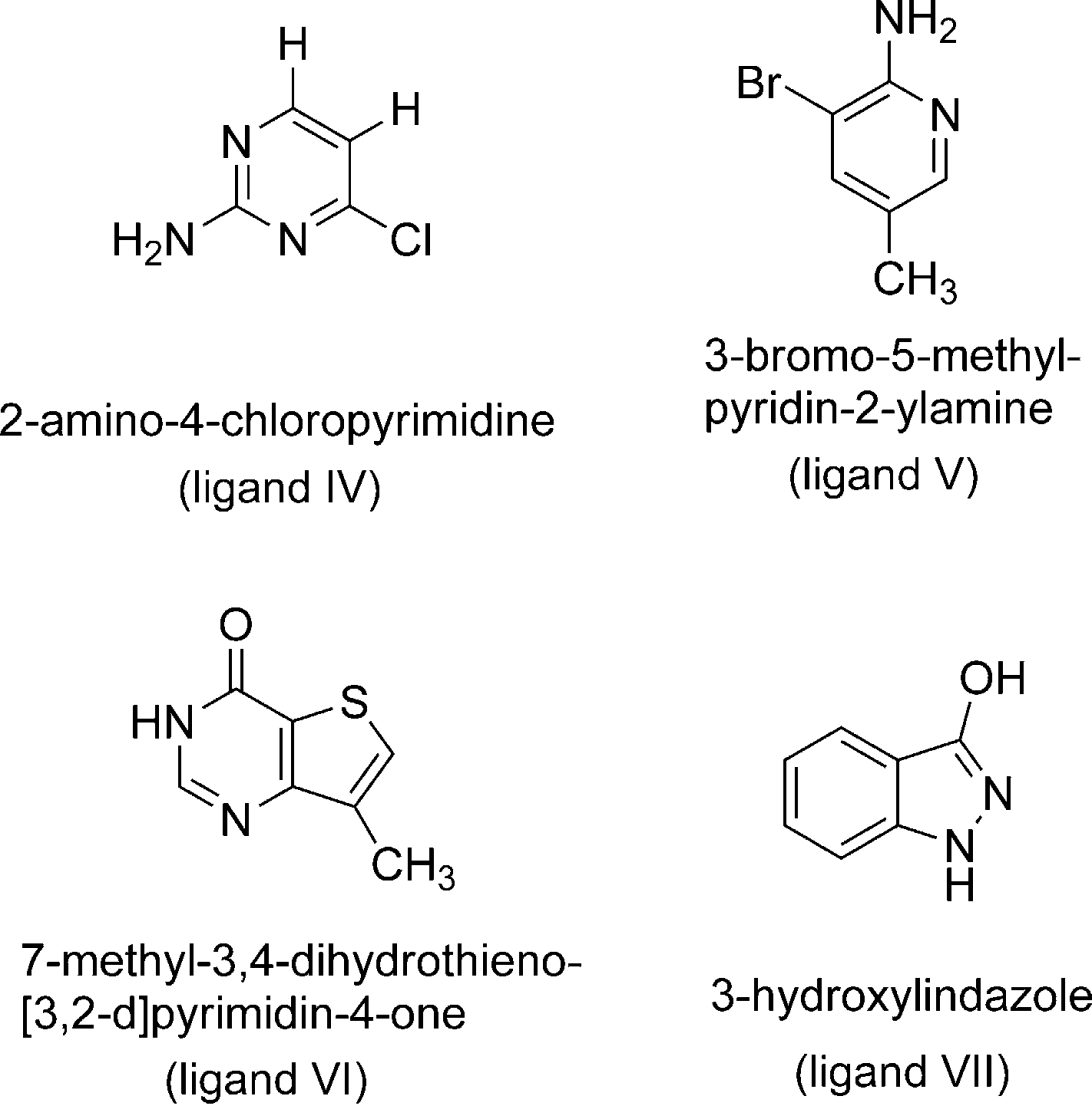
Fragments identified by LLS as weak binders for Hsp90. Their dissociation constants *K*_D_ were measured for the first time by LLS, as described in the text and Table 3.

In the absence of competing binders, the interaction between the spy ligand and the protein leads to rapid LLS relaxation and hence to the attenuation of the LLS signal (spectrum 1 in Figure 4); conversely, the presence of a competitor leads to a partial displacement of the spy ligand, hence to slower LLS relaxation and a partial restoration of the LLS signal of the spy (spectrum 2 in Figure 4). This change in LLS signal is due to a mere 13 % change in the amount of bound ligand, which itself is only 0.3 % of the total ligand concentration.

**Figure 4:**
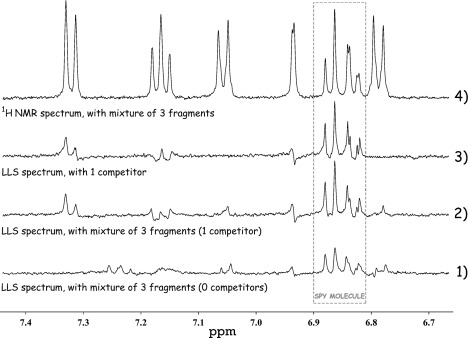
Identification of a weak binder in a mixture. 1) Weak LLS signals of the spy ligand after sustaining the LLS for Δ=2.5 s in the absence of a competing binder in mixture 1 (spy ligand [II]=500 μm with *K*_D_=790 μm protein [Hsp90]=2.5 μm and three nonbinding ligands: 600 μm tyrosine, 600 μm 3,4-difluorobenzylamine, and 600 μm 4-trifluoromethylbenzamidine). 2) Enhanced LLS signals in the presence of a weak binder (mixture 2 contains 600 μm of the weakly binding ligand [V] 3-bromo-5-methylpyridin-2-ylamine (*K*_D_=2.2 mm), instead of 600 μm of the nonbinding ligand 3,4-difluorobenzylamine). 3) LLS signals observed in the presence of only the binding fragment (mixture 3 contains 500 μm spy ligand [II], 2.5 μm protein [Hsp90], and 600 μm of the weakly binding ligand [V] 3-bromo-5-methylpyridin-2-ylamine). 4) Conventional ^1^H NMR spectrum of mixture 2.

Once the presence of a binder in a mixture has been demonstrated, a deconvolution step is needed to identify the hit, as shown in Figure 4, which allowed the identification of 3-hydroxyindazole as a weak binder for Hsp90.

Note that to displace a weak spy ligand by even more weakly binding fragments, the latter must be present at similar concentrations. This is a considerable advantage over other NMR methods used in competition mode, which because of the lower contrast require much higher protein concentrations to achieve effective displacements, as they require small ligand/protein ratios in order to detect weak ligands.

Furthermore, if the mixtures comprise many components, the NMR resonances of the spy molecule may be obscured by overlapping signals,[18] which may hamper other NMR methods when used in competition mode. Fortunately, the LLS sequence in effect eliminates signals that do not stem from long-lived states. As shown by spectrum 2 in Figure 4, resonances that arise from other compounds are considerably reduced, compared to the conventional ^1^H spectrum of the same mixture (spectrum 4). The problem could be further reduced by selecting a spy ligand with a larger difference in chemical shifts, in order to decrease the likelihood that the two doublets stemming from the LLS overlap with other signals.

Once weak binders have been identified, their dissociation constants *K*_D_ can be determined from 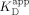 of the spy molecule upon titration of the spy ligand in the presence of a constant amount of a weak binder or vice versa.[15] Titration of a spy ligand makes it possible to use the same experimental setup for different fragments. The highest concentrations of the competing ligands are limited only by their aqueous solubility. At each concentration, the rates *R*_LLS_ can be obtained from the ratio of LLS signal intensities observed with two different sustaining delays *τ*_a_ and *τ*_b_. In order to verify that our procedures are self-consistent, the dissociation constant *K*_D_ of ADP (ligand I) was determined by monitoring *R*_LLS_ in vanillic acid diethylamide (ligand II) while titrating 500 μm<[L_II_]<5 mm with 10 μm Hsp90 and a fixed ADP concentration [L_I_]=15 μm. The resulting *K*_D_(L_I_)=8±3 μm is in reasonable agreement with *K*_D_(L_I_)=15±10 μm determined by direct titration of ADP (ligand I) against Hsp90 (Table 2). Following a similar procedure, the affinity of 2-aminopyrimidine (ligand III) (Figure 5) was determined with 10 μm Hsp90, using a fixed concentration [L_III_]=7 mm, and titrating 500 μm<[L_II_]<5 mm. The fitted dissociation constant *K*_D_(L_III_)=11±2 mm suggests very weak binding of this ligand to the protein, which must, however, clearly be specific to explain these observations. The fragment bound to Hsp90 was also observed by X-ray crystallography, as reported by Murray et al.[16]

**Figure 5:**
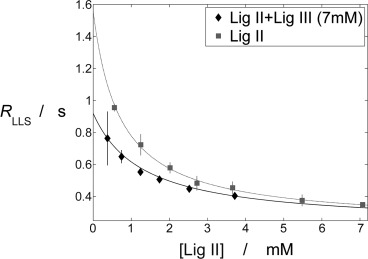
Gray line: Direct titration of vanillic acid diethylamide (ligand II) in the presence of 10 μm Hsp90 protein. Black line: Competition experiment with titration of vanillic acid diethylamide (ligand II) as a spy ligand in the presence of 10 μm Hsp90 protein and a constant concentration of 7 mm of 2-aminopyrimidine (ligand III).

This approach was used to measure the dissociation constants *K*_D_ of the four fragments shown in Figure 2. The values derived from full titrations and from experiments with a single concentration are in good agreement (Table 3).The latter procedure is to be preferred when fragment hits are ranked according to affinity after a screening campaign.

**Table 3:** Dissociation constants of fragments measured by LLS competition binding experiments.

Ligand	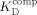 [mm]	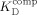 [mm] (from first point of titration)	Concentration [L] of competing ligands (mM)
I	0.008±0.003	0.010	0.015
II	(spy)	–	–
III	11±2	12	7
IV	0.9±0.2	0.7	0.9
V	1.4±0.9	2.2	1
VI	1.7±0.9	2.2	1.6
VII	7±1	5	2.9

Note that the choice of the spy molecule determines the experimental conditions of the LLS assay. With our choice of a spy molecule (ligand II, *K*_D_=790 μm), a concentration of 7 mm of the competing ligand III (*K*_D_=12 mm) gives rise to 19 % contrast (first point of black curve in Figure 5). This can be reduced to 3.3 mm to give rise to a 10 % contrast, which is sufficient to show binding in screening experiments, as shown in Figure 4 for ligand V (difference between spectra 1 and 2). If the expected affinities of fragments for a particular target are on the order of *K*_D_=5 mm or higher, it is most convenient to identify and use a weaker spy molecule that would ensure a 10 % contrast while working at lower fragment concentrations [see Eq. (4)]. As a consequence, one can effectively screen and identify weak fragments with very low solubility.

In summary, the detection of LLS requires ligands that contain a (preferably isolated) two-spin system, a condition that cannot easily be fulfilled for all fragments in a screening “cocktail”. We have therefore shown that the LLS method can be used very effectively in a competitive approach, where the displacement of a suitable spy molecule is detected through the effect on its LLS. We have demonstrated that such LLS-filtered competition experiments can be used to screen and determine the binding constants of very weakly binding fragments with *K*_D_ up to 12 mm by using ligand/protein ratios as large as 200. This advantage can be put to good use by reducing the concentration of the target protein, thus extending the sensitivity of detection compared to established NMR screening methods like LOGSY, STD, and *T*_1*ρ*_. A sufficient contrast can be achieved with ligand/protein ratios up to 700, which makes it possible to detect the binding of fragments with *K*_D_=790 μm. Alternatively, the ability to detect the presence of very small concentrations of protein–ligand complexes can be used to determine very weak binding (*K*_D_>10 mm), which facilitates the screening of fragments for challenging protein targets. We have also shown that we can quantitatively determine the dissociation constants of a spy molecule and various competing ligands. The ability to measure accurate binding constants in the mm range, where methods such ITC and high concentration assays may fail, in particular when the ligand solubility is limited, enables the investigation of structure–activity relationships and the guidance of initial steps of hit-optimization chemistry.

## Notes

Supporting information for this article (including details on the experiments and sample preparation) are described in the supporting informationis available on the WWW under http://dx.doi.org/10.1002/anie.201404921.
