# Supplementary material for: Exploring Weak Ligand–Protein Interactions by Long-Lived NMR States: Improved Contrast in Fragment-Based Drug Screening
Source: Angew Chem Int Ed Engl. 2014 Sep 4;53(42):11376–80. doi: 10.1002/anie.201404921 (PMC4497601; doi:10.1002/anie.201404921)
Supplement: Supplementary file 1 [file anie0053-11376-sd1.pdf]

Angewandte Chemie
Eine Zeitschrift der Gesellschaft Deutscher Chemiker

Supporting Information
© Wiley-VCH 2014
69451 Weinheim, Germany

Exploring Weak Ligand–Protein Interactions by Long-Lived NMR States: Improved Contrast in Fragment-Based Drug Screening**

Roberto Buratto, Daniele Mammoli, Elisabetta Chiarparin, Glyn Williams, and Geoffrey Bodenhausen*

anie_201404921_sm_miscellaneous_information.pdf

Contents

1. Sample preparation..2

2. Experimental procedures..2

3. LLS titration of a spy molecule..3

4. Fitting procedure..3

1. Sample preparation

All solutions for screening and titration experiments were prepared in 20 mM TRIS buffer with 150 mM NaCl, 5 mM $MgCl_2$, and 1 mM TCEP. Stock solutions were prepared in d6-DMSO containing 150 mM vanillic acid diethylamide (Sigma-Aldrich, purity >98%), 150 mM 2-amino-4-chloro-pyrimidine (Fluorochem, purity > 97%), 150 mM 2-aminopyrimidine (Acros, purity 98%), 400 mM 3-hydroxyindazole, 400 mM 7-methyl-3,4-dihydrothieno[3,2-d]pyrimidine-4-one and 400 mM 3-bromo-5-methyl-pyridine-2-ylamine from Astex' fragment library. For ADP (Sigma-Aldrich, as sodium salt hydrate from bacterial source, purity > 95 %), a 150 mM stock solution was prepared in 20 mM TRIS buffer with 150 mM NaCl, 5 mM $MgCl_2$, and 1 mM TCEP. A hexahistidine-tagged N-terminal fragment (9-224) of HSP90α was cloned into a pET28 vector and expressed in E.Coli BL21 (DE3). The expressed protein was retained on a Ni^{2+} affinity column, followed by cleavage of the His tag using thrombin and purification on a Superdex75 gel filtration column. The final buffer used contained 20 mM TRIS-HCl, pH7.4, 150 mM NaCl with 1 mM β-ME.

2. Experimental procedures

The direct titration of ADP (ligand I) against Hsp90 was performed by adding 2-5 µL aliquots of ADP stock solution to 400 µL of a 10 µM solution of Hsp90. Titrations of ligands II and IV were carried out with the same procedure. Competition titrations were performed by addition of 2-5 µL aliquots of the stock solution of ligand II, on the one hand, to 400 µL of a 10 µM solution of Hsp90 with 15 µM ADP, and, on the other hand, to 400 µL of a 10 µM solution of Hsp90 containing 100 µM of ligand IV. The same procedure was followed to determine the K_D of the ligands V, VI and VII: the first one was performed in the presence of [ligand V] = 1 mM, the second one in the presence of [ligand VI] = 1 mM and the last one in the presence of [ligand VII] = 2.92 mM. All NMR measurements were performed on a 500 MHz (11.74 T) spectrometer equipped with a cryoprobe at 25°C. The experiments were performed with the following pulse sequence for the excitation, sustaining and observation of long-lived states (LLS) [1]:

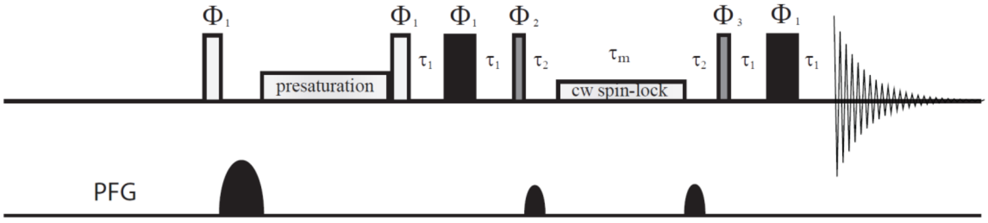

Figure 1 Pulse sequence to determine relaxation rates R_{LLS} of long-lived states (LLS).

where pulses with flip angles of $\pi/2$, $\pi/4$ (45°) and π are indicated by white, grey and black rectangles, respectively. For a system comprising two non-equivalents spins I = ½ and S = ½ with a chemical shift difference $\Delta\nu_{IS} = (\Omega_I - \Omega_s)/2\pi$ and a scalar coupling constant J_{IS}, the intervals τ_1 and τ_2 must be set to:

$$\tau_1 = (4\,J_{IS})^{-1} \qquad (1)$$

$$\tau_2 = (2\,\Delta\nu_{IS})^{-1} \qquad (2)$$

At the beginning of the experiment, a π/2 pulse followed by a pulsed field gradient along the z axis were applied to suppress the magnetization. A weak water presaturation pulse lasting 3 s was applied in the subsequent recovery delay. During the sustaining delay 1.2 s < τ_m < 3.5 s (chosen depending on the expected the constant $T_{LLS} = 1/R_{LLS}$), continuous-wave radio-frequency (rf) irradiation was applied, with the carrier set half-way between the two chemical

shifts, $\Omega_{carrier} = (\Omega_I+\Omega_S)/2$, in order to make the spins magnetically equivalent in the sense of average Hamiltonian theory. Two pulse field gradients were applied during the two τ_2 delays in order to dephase double quantum coherences. The acquisition time was 2.2 s. The phase cycle was Φ_1 = (x,-x), Φ_2 = (y,y,-y,-y), Φ_3 = (y) and $\Phi_{receiver}$ = (x,-x,-x,x). The number of scans was chosen according to the concentration. For example, during screening experiments with 500 µM of spy ligand, 64 scans were accumulated, thus requiring 10 minutes per sample. The rates R_{LLS} were obtained from the ratio of the signal intensities observed using two different sustaining delays τ_a and τ_b, repeating each of them twice in order to compare four pairs of signal intensities $I_a(\tau_a)$ - $I_b(\tau_a)$:

$$R_{LLS} = \frac{log\frac{I_a}{I_b}}{(\tau_b - \tau_a)} \qquad (3)$$

Typically, we used τ_a = 0.5 s and τ_b in the vicinity of the estimated value of $T_{LLS} = 1/R_{LLS}$.

3. LLS titration of a spy molecule

The LLS titration of the spy molecule without competitor was carried out by addition of aliquots of a stock solution of 150 mM vanillic acid diethylamide in DMSO to 400 µL of a 10 µM solution of Hsp90 protein in the buffer described above. The contrast C_{LLS} was determined after sustaining the LLS for τ_a = 0.5 s and 1.0 < τ_b < 2.5 s.

Vol added / µL	[protein] / µM	$[L]_{tot}/[P]_{tot}$	X^{bound}	Contrast C_{LLS} / %
1.50	10.00	56	0.74	72
3.30	9.96	125	0.49	63
5.40	9.90	202	0.36	54
7.20	9.86	272	0.28	45
9.70	9.80	366	0.22	41
14.50	9.69	548	0.16	29
18.80	9.59	707	0.13	23

4. Fitting procedure

The relaxation rates R_{LLS}^{obs} measured during the titrations were fitted to the following equation:

$$R_{LLS}^{obs} = \frac{[PL]}{[L_{tot}]} R_{LLS}^{bound} - (1 - \frac{[PL]}{[L_{tot}]}) R_{LLS}^{free} \qquad (4)$$

where $$\frac{[PL]}{[L_{tot}]} = X_{bound} = \frac{[P_{tot}]+[L_{tot}]+K_D-\sqrt{([P_{tot}]+[L_{tot}]+K_D)^2-4[P_{tot}][L_{tot}]}}{2[L_{tot}]} \qquad (5)$$

By fitting the relaxation rates R_{LLS}^{obs} to equation 5, one can determine the dissociation constant K_D. One should perform two kinds of titration experiments:

Direct titration. If the spy ligand that carries the LLS is titrated against the protein in the absence of any competitors, one obtains $K_D = K_{D,\ true}^{spy}$, i.e., is the true value of the dissociation constant of the spy ligand.

Competition titration. If the spy molecule is titrated against the protein in the presence of a competitor, one initially obtains $K_D = K_{D,\ app}^{spy}$ the apparent dissociation constant of the spy ligand. Note that $K_{D,\ app}^{spy} > K_{D,\ true}^{spy}$. The latter can be extracted by LLS titration without competitor, or by using non-NMR methods such as ITC.

One may calculate the dissociation constant K_D^{comp} of the competitor with the following relationship [2]:

$$K_D^{comp} = \frac{[L^{comp}]K_{D,\ true}^{spy}}{K_{D,\ app}^{spy} - K_{D,\ true}^{spy}} \qquad (6)$$

where K_D^{comp} is the dissociation constant of the competitor and $[L^{comp}]$ is the concentration of the competitor.

The errors (root-mean-square deviations) were estimated from the four pairs of signal intensities obtained using two different sustaining delays τ_a and τ_b and repeating each experiment twice.

[1] R. Sarkar, P. R. Vasos, G. Bodenhausen, *J Am Chem Soc* **2007**, *129*, 328-334.

[2] C. Dalvit, M. Flocco, S. Knapp, M. Mostardini, R. Perego, B. J. Stockman, M. Veronesi, M. Varasi, *J Am Chem Soc* **2002**, *124*, 7702-7709.
